# Long-Term Survival of Endometriosis-Related Ovarian Clear Cell Carcinoma with Endometriosis Surgical History

**DOI:** 10.3390/jcm14051550

**Published:** 2025-02-26

**Authors:** Yun Soo Chung, Jin Kyung Baek, Euna Choi, Hae-Rim Kim, Heeyon Kim, Yong Jae Lee, Bo Hyon Yun, Seok Kyo Seo

**Affiliations:** 1 Department of Obstetrics and Gynecology, Severance Hospital, Yonsei University College of Medicine, Seoul 03722, Republic of Korea; espera88@yuhs.ac (Y.S.C.); nupy90@yuhs.ac (J.K.B.); euna_ya@yuhs.ac (E.C.); kimhy@yuhs.ac (H.K.); svass@yuhs.ac (Y.J.L.); garfieldzz@yuhs.ac (B.H.Y.); 2 Department of Statistics, University of Seoul, Seoul 03722, Republic of Korea; haley2203@naver.com

**Keywords:** endometriosis, ovarian clear cell carcinoma, endometriotic ovarian cyst surgery, survival, CA-125

## Abstract

**Background/Objectives**: The prognosis of endometriosis-related ovarian clear cell carcinoma (OCCC) versus non-endometriosis-associated OCCC remains unclear. We examined the impact of endometriosis on OCCC diagnosis and progression and assessed whether prior surgical intervention for endometriotic ovarian cysts affects prognosis. **Methods**: In this retrospective study (2006–2024), OCCC patients were classified as non-endometriosis-associated or endometriosis-related. A subgroup analysis compared endometriosis-related OCCC patients with and without a history of endometriotic ovarian cyst surgery. **Results**: The average CA-125 level was 104.20 (29.90, 347.70) in the non-endometriosis-associated OCCC group and 80.70 (32.40, 247.90) in the endometriosis-related OCCC group (*p* = 0.32). Early-stage diagnosis occurred in 62.77% and 75.21% of these groups, respectively (*p* = 0.046). The average age at diagnosis was 53.95 ± 9.71 years for the non-endometriosis-associated group and 45.68 ± 7.98 years for the endometriosis-related group (*p* < 0.001). Mortality or poor prognosis was observed in 24.11% and 17.80% of these groups, respectively (*p* = 0.226). In endometriosis-related OCCC, comparisons were made between patients with and without a history of endometriotic ovarian cyst surgery. The average age at diagnosis was 45.84 ± 8.24 years for those without a surgical history and 44.71 ± 6.35 years for those with a surgical history (*p* = 0.59). Early-stage diagnosis was observed in 77.23% and 62.50%, respectively (*p* = 0.339). Mortality or poor prognosis occurred in 14.85% of those without a surgical history and 35.29% of those with a surgical history (*p* = 0.008). The hazard ratio for women with a surgical history was 3.48 (1.29–8.69) (*p* = 0.008). The incidence rate was 3.17 per 1000 person-years (PYRs) for individuals without surgery and 13.36 per 1000 PYRs for those with a history of surgical intervention (*p* = 0.008). **Conclusions**: Endometriosis did not impact the prognosis of women with OCCC. However, women with endometriosis-related OCCC were diagnosed at earlier stages and at younger ages. A history of endometriotic ovarian cyst surgery did not influence OCCC detection but was linked to poorer survival outcomes.

## 1. Introduction

Multiple hypotheses have been proposed to explain the etiology of endometriosis. The most compelling explanation is the growth of endometrial glands and stroma outside the uterine cavity [1,2,3]. Endometriosis itself is a benign condition, but oxidative stress in endometriotic tissues and alterations in the microenvironment at the ectopic location can trigger malignant changes [4,5]. Malignant transformation in endometriosis was first proposed by Sampson in 1925 [5,6]. Furthermore, numerous studies have investigated the association between endometriosis and the incidence of ovarian clear cell carcinoma (OCCC) [7]. Some studies indicate that specific genes, such as p53, KRAS, PTEN, and ARID1A, are involved in the transition from endometriosis to invasive carcinomas [8]. The probability of developing ovarian cancer in women with endometriosis is approximately 0.3–0.8%, which is 2–3 times higher than the probability among those without endometriosis [1]. Specifically, the risk of developing clear cell or endometrioid histotypes is significantly higher than that of other histotypes [9].

The prognosis of endometriosis-related OCCC compared with that of non-endometriosis-associated OCCC remains controversial. Some studies have suggested that individuals with OCCC associated with endometriosis are diagnosed at a younger age and at earlier stages (Stage I/II), leading to better prognoses [7,10,11,12,13]. Some studies have reported that, due to the nature of endometriosis, clear cell carcinomas arising from it tend to occur in younger patients, with unilateral involvement and a reduced presence of ascites. However, these studies did not consistently associate endometriosis with a lower-stage tumor or better prognosis [14]. The variability in prognostic outcomes may be attributed to differences in study criteria, including age, the FIGO stage, tumor laterality, and the presence of ascites. Furthermore, some studies suggest that the improved progression-free survival and overall survival in these patients are primarily linked to early-stage detection and optimal debulking surgery, rather than the direct impact of endometriosis itself [15]. For example, reference [14] demonstrated a favorable prognostic role of endometriosis, but this was largely attributed to the earlier diagnosis of OCCC at early stages. Similarly, another study found that the observed favorable prognosis in endometriosis-associated OCCC is more likely due to a higher proportion of stage I and II cases rather than a direct survival advantage conferred by endometriosis [16].

Melin et al. reported that oophorectomy, along with the removal of all visible endometriosis, may provide a protective effect against future ovarian cancer development [17]. Additionally, some studies recommend that treatment strategies for endometriosis should be tailored based on the patient’s reproductive plans, family planning goals, and family history [18]. In cases where future fertility is not a concern, bilateral salpingectomy may be considered to reduce the risk of high-grade serous carcinoma [18]. Furthermore, one study suggested that emergency surgery following the rupture of an endometriotic ovarian cyst may lead to a better prognosis, particularly in patients without a history of prior surgical interventions for endometriosis [19]. While some research has explored the benefits of surgery in reducing malignant transformation risk and the potential impact of previous surgeries on the prognosis of ruptured endometriomas [19], no studies have been identified that specifically evaluate the prognosis of women with a history of surgical intervention for endometriotic ovarian cysts among those diagnosed with OCCC. Therefore, this study aimed to elaborate on the survival outcomes and clinical characteristics of women in two groups: one including those with OCCC related to endometriosis who had not undergone surgical procedures and the other including those who had undergone surgical procedures for endometriotic ovarian cysts.

## 2. Materials and Methods

### 2.1. Study Design, Data Collection, and Participants

This retrospective, single-institution analysis was conducted at Severance Hospital, Seoul, Republic of Korea. This study included patients histologically diagnosed with OCCC between 2006 and 2024; among them, two groups were identified based on pathological findings: those with only clear cell carcinoma and those with both clear cell carcinoma and endometriosis. A subgroup analysis of women with OCCC and endometriosis was conducted based on their surgical histories related to endometriotic ovarian cysts.

The inclusion criteria for this study were patients pathologically diagnosed with OCCC, without restrictions based on other medical histories, stages of endometriosis, or performance status. Patients were included regardless of their age, comorbidities, or treatment history. The exclusion criteria were patients lacking sufficient medical records to confirm any surgical history related to endometriosis or OCCC. No specific performance status or endometriosis staging criteria were applied as exclusion parameters, reflecting the broad nature of this retrospective study.

Ethical approval for data collection was obtained from the Ethics Committee of Yonsei University Institutional Review Board (4-2024-0737).

### 2.2. Statistical Analysis

Descriptive statistics were used to compare the clinical features of women with and without endometriosis-related OCCC. The variables analyzed included age, the stage of OCCC, cancer grade, ovarian mass location at diagnosis, a history of breast and endometrial cancer, and residual disease after staging surgery. CA-125 was included as it is a commonly used biomarker for ovarian cancer screening and monitoring [20,21,22]. The prognosis of OCCC was evaluated by comparing expired cases and hopelessly discharged cases. Stages I and II were classified as early-stage cancers, while stages III and above were categorized as advanced-stage cancers [22]. The pathological diagnosis of cancer grade was determined based on the following criteria: Grade 1: nearly normal tissue; Grade 2: some loosely packed abnormal cells; Grade 3: many abnormal cells; Grade 4: very few normal cells; and Grade 5: no normal cells. Residual disease was documented by the surgeon in the operative notes.

Age and CA-125 levels were treated as continuous variables, whereas stage, cancer grade, ovarian mass location, breast cancer history, endometrial cancer history, and residual disease were treated as categorical variables. Since the age variable satisfied the assumption of normality, a *t*-test was used. Age was reported as means and standard deviations. For CA-125, the distribution did not meet the normality assumption; therefore, the Wilcoxon test, a non-parametric statistical method, was applied, and the results were presented as medians and interquartile ranges.

Categorical variables, including stage, cancer grade, ovarian mass location, breast cancer history, endometrial cancer history, residual disease, and prognosis (terminal or expired status), were compared using the chi-squared test to evaluate the significance of differences among the groups. These variables were presented as percentages and absolute numbers.

In this study, survival analysis was conducted by counting expired or hopelessly discharged cases, as these patients were in terminal stages. Patients with terminal-stage disease were discharged because no further treatments were deemed effective. Survival analysis comparing the two groups was performed using the Cox proportional hazards model. Survival curves were estimated for each group separately using the Kaplan–Meier method and statistically compared using the log-rank test. The proportional hazards assumption was tested based on the Schoenfeld residuals. In the case of loss to follow-up, the evaluation was considered complete up to the point when the patient stopped visiting the hospital. Since the data met the proportional hazards assumption, the Cox proportional hazards model and the Kaplan–Meier curve were used to analyze the trend in patient prognosis. Statistical significance was set at *p* < 0.05. All statistical analyses were performed using R software version 4.3.1 (The R Foundation, www.R-project.org).

## 3. Results

### 3.1. Characteristics of Participants

**Figure 1:**
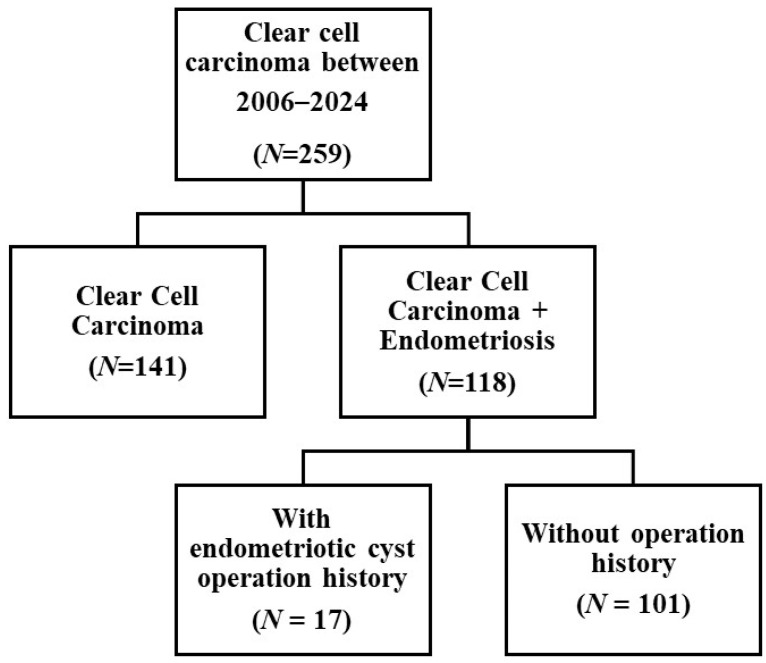
Diagram of the study population.

Between 2006 and 2024, 259 women diagnosed with OCCC were treated at Severance Hospital in Seoul, Republic of Korea. Based on histopathology, the participants were divided into two groups: 141 women with non-endometriosis-associated OCCC and 118 women with both OCCC and endometriosis (Figure 1).

The basic clinical characteristics of the study participants are presented in Table 1. All participants in this study were of Asian ethnicity. While ethnicity was not a variable of primary focus, it is acknowledged as a potential factor in disease presentation and outcomes. The only statistically significant differences between the two groups were the average age and stage at diagnosis. Women with endometriosis-related OCCC were diagnosed at an average age of 45.68 ± 7.98 years, whereas those with non-endometriosis-associated OCCC were diagnosed at an average age of 53.95 ± 9.71 years (*p* < 0.001) (Table 1).

Among women with both OCCC and endometriosis, only 17 had a history of surgical intervention for endometriotic ovarian cysts (Figure 1). The clinical characteristics of the patients are presented in Table 2. Most patients with a history of surgery had their initial procedure performed at another institute and were referred to our institute upon the detection of recurrence of ovarian cysts. Since all participants underwent surgery for endometrioma and previous research has reported that endometriomas are a form of deep infiltrating endometriosis typically associated with at least stage III endometriosis [23], it is assumed that most had endometriosis at stage III or higher based on surgical findings and their clinical history. Among the cohort, two patients who had surgery at Severance Hospital were pathologically diagnosed with stage IV endometriosis. No significant differences were observed between the patients with and without a history of endometriotic ovarian cyst surgery (Table 2).

### 3.2. OCCC Coexisting with Endometriosis

Women with and without endometriosis were compared to evaluate the association between endometriosis and ovarian carcinoma. Among the participants, 141 women had only clear cell compartments, whereas 118 women had both endometriosis and clear cell compartments. The median CA-125 levels were compared between groups using the Wilcoxon test. In the non-endometriosis-associated OCCC group, the median CA-125 level was 104.20 (IQR: 29.90, 347.70), while in the endometriosis-related OCCC group, it was 80.70 (IQR: 32.40, 247.90) (*p* = 0.32) (Table 3). No statistically significant difference was observed between the two groups.

When comparing the proportion of early-stage (Stage I/II) and late-stage (Stage III/IV) non-endometriosis-associated OCCC and endometriosis-related OCCC, 62.77% of women in the non-endometriosis-associated OCCC group were in the early stages, compared to 75.21% in the endometriosis-related OCCC group (*p* = 0.046) (Table 1). This finding suggests a significantly higher incidence of Stage I/II endometriosis-related OCCC (*p* = 0.046) (Table 1).

To compare the prognosis between the non-endometriosis-associated OCCC and endometriosis-related OCCC groups, we counted the number of women who died or were hopelessly discharged from the hospital as being in terminal stages: in the non-endometriosis-associated OCCC group, 24.11% of the women; in the endometriosis-related OCCC group, 17.80% (*p* = 0.226) (Table 3). The hazard ratio for endometriosis-related OCCC compared to the non-endometriosis-associated OCCC group was 0.71 (95% confidence interval: 0.42–1.23; *p* = 0.226) (Table 3). Since the confidence interval includes one, this indicates that there is no significant difference in prognosis between the two groups.

**Figure 2:**
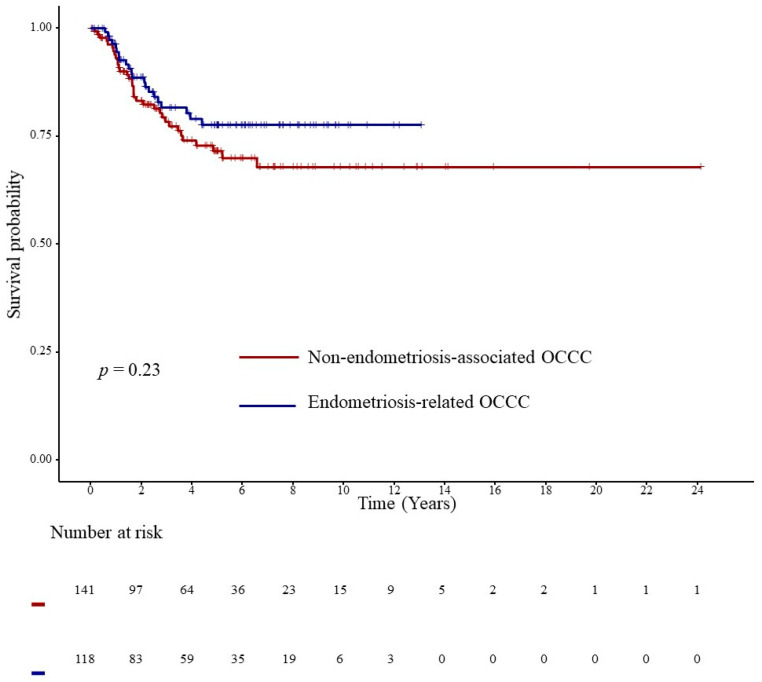
Kaplan–Meier curve for non-endometriosis-associated OCCC and endometriosis-related OCCC. OCCC, ovarian clear cell carcinoma.

A survival analysis of endometriosis and OCCC was conducted using Kaplan–Meier curves and the Cox proportional hazards model, which revealed no significant differences between non-endometriosis-associated OCCC and endometriosis-related OCCC (*p* = 0.23) (Figure 2).

### 3.3. Surgical Intervention History of Endometriotic Ovarian Cysts

To assess the impact of surgical intervention for endometriotic ovarian cysts on endometriosis-related OCCC, we compared CA-125 levels and the stage at diagnosis. CA-125 levels at diagnosis were compared between women with endometriosis-related OCCC with and without a history of surgical intervention for endometriotic ovarian cysts. Among those with endometriosis-related OCCC, 101 had no history of surgical intervention, and only 17 had a history of surgical intervention (average duration between previous surgery and the detection of OCCC, 8.522 years) (Table 4). There were no statistically significant differences in CA-125 levels, as determined using the Wilcoxon test [77.40 (IQR: 32.10, 241.60) vs. 84.00 (IQR: 40.80, 259.35), *p* = 0.629] (Table 4).

Among women with endometriosis-related OCCC and no history of surgical intervention for endometriotic ovarian cysts, 77.23% were diagnosed at an early stage (Stage I/II) compared to 62.5% of those with a surgical history (*p* = 0.339) (Table 2).

The prognosis was compared between women with endometriosis-related OCCC with and without a history of surgical intervention for endometriotic ovarian cysts by counting the number of cases of expiration or hopeless discharge. A significantly higher proportion of women with a history of surgery (35.29%) experienced either death or hopeless discharge than those without (14.85%) (*p* = 0.008) (Table 4).

**Figure 3:**
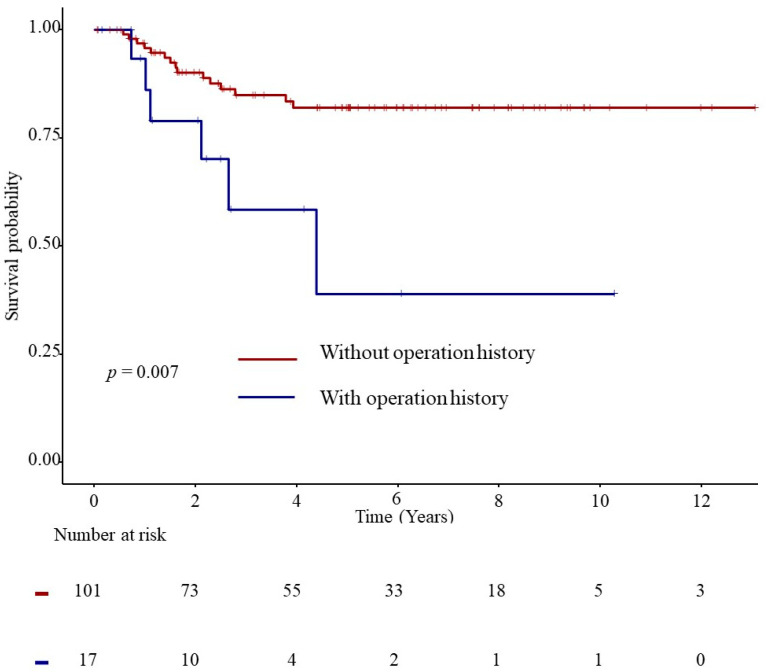
Kaplan–Meier curve for women with endometriosis-related OCCC and a history of surgical intervention for endometriotic ovarian cysts and those without a surgical history. OCCC, ovarian clear cell carcinoma.

A survival analysis of women with endometriosis-related OCCC with and without surgical intervention for endometriotic ovarian cysts was conducted using Kaplan–Meier curves and the Cox proportional hazards model. The analysis revealed that women who underwent surgical intervention had a lower survival probability than those who did not (*p* = 0.007) (Figure 3). The hazard ratio with a 95% confidence interval for women with endometriosis-related OCCC and a history of surgical intervention for endometriotic ovarian cysts was 3.48 (1.39–8.69) (*p* = 0.008) (Table 4).

## 4. Discussion

Although endometriosis is a benign condition, its characteristics resemble those of malignancy [4]. The mechanisms underlying the malignant transformation from endometriosis to clear cell carcinoma remain unclear [24]; however, several microenvironmental factors essential for malignant transformation, such as oxidative stress, immune cell dysfunction, inflammation, and steroid hormones, have been identified [25]. The aim of postoperative hormonal treatment is to prevent malignant transformation or the recurrence of ovarian endometriomas after surgical treatment [26], but the relationship between endometriosis and survival rate in OCCC remains controversial.

In the current study, the endometriosis-related OCCC group was diagnosed at an earlier stage and at a younger age compared with the non-endometriosis-associated OCCC group, unlike other types of ovarian cancer, which are typically diagnosed at an advanced stage [24]. Previous studies have suggested that endometriosis-related OCCC is often diagnosed at an early stage because of characteristic symptoms such as pelvic pain, dysmenorrhea, and dyspareunia [10,24,27,28,29,30]. Some studies suggest that because endometriosis-related OCCC is often detected at an earlier stage and at a younger age, endometriosis may be an independent prognostic factor for OCCC [11]. Despite this early diagnosis, the overall prognosis of endometriosis-related OCCC is not better than that of non-endometriosis-associated OCCC.

There are several possible explanations for these results. At advanced stages, endometriotic tissues may become scarce or even absent owing to complete malignant transformation [5,6]. Therefore, non-endometriosis-associated OCCC may also originate from endometriosis, with no significant histological differences observed between endometriosis-related OCCC and non-endometriosis-associated OCCC. Another explanation is that, rather than endometriosis, the status of residual tumors following cytoreductive surgery may primarily influence disease prognosis [15,24,29]. Although endometriosis may contribute to malignant transformation, once malignancy is established, it does not affect the prognosis [4,13,31].

Many studies have emphasized that optimal cytoreductive surgery and early detection are crucial for the prognosis of endometriosis-related OCCC [15]. Patients with a history of endometriotic ovarian cyst surgery often experience pelvic pain, dysmenorrhea, and dyspareunia, which may lead them to become accustomed to pain and delay seeking appropriate treatment, ultimately resulting in a poorer prognosis. Another hypothesis supporting the poor prognosis of patients with a surgical history is the formation of adhesions after surgery. Previous research has shown that, although the likelihood of adhesion formation varies between surgical specialties and procedures, the incidence of pelvic adhesions ranged from 25% to 92% [32]. Although there is no objective parameter to compare adhesion levels during the staging operation in this study, it is possible that previous surgical interventions may have led to severe adhesions or anatomical changes, making it difficult to recognize residual disease. This could explain the lack of differences in residual disease between the groups while still contributing to incomplete tumor resection in subsequent procedures, ultimately affecting prognosis. Another hypothesis is related to the microenvironment of endometriosis, which includes decreased natural killer cell (NK cell) activity and increased transforming growth factor-β1 (TGF-β1) activity [33]. Badawy et al. reported that the chocolate fluid in endometriotic cysts is enriched with TGF-β1 and is associated with decreased NK cell activity [33]. While some studies suggest that TGF-β2, rather than TGF-β1, is a biomarker for poor prognosis in ovarian cancer [34], Liu et al. found that TGF-β1 may increase resistance to apoptosis in endometriotic tissue, promote survival by decreasing immune cell activity, and facilitate cell migration and invasion [35]. Based on these findings, previous surgery could result in the spillage of endometriotic ovarian cyst fluid, which increases TGF-β1 levels and decreases NK cell activity, ultimately contributing to a poor prognosis.

Ovarian endometrioma is the most common form of endometriosis, occurring in approximately 17–46% of cases where endometriosis is diagnosed. While the malignant transformation of endometriomas is rare, occurring in less than 1% of cases, it remains a significant concern [36,37]. This underscores the importance of long-term monitoring and early detection strategies in patients with endometriosis. Since individuals with endometriotic ovarian cysts are exposed to microenvironmental factors, such as oxidative stress, immune cell dysfunction, inflammation, and steroid hormones, which are essential for malignant transformation [25], understanding the molecular biology underlying endometriosis-related OCCC could shed light on the transformation from endometriosis to OCCC and its prognosis. In this study, CA-125 was utilized to compare the groups; however, emerging research suggests that newer biomarkers, such as circulating tumor DNA, extracellular vesicles, and non-coding RNAs, may enhance the differential diagnosis of ovarian neoplasia [36]. Incorporating these novel biomarkers into future studies could improve the predictive accuracy of ovarian cancer risk in patients with a history of endometriosis and endometriotic ovarian surgery.

Furthermore, studies have suggested that oophorectomy and the complete removal of visible endometriotic lesions may provide a protective effect against ovarian cancer development [17]. This protective effect may be linked to alterations in the local inflammatory and hormonal microenvironment surrounding endometriotic lesions. Investigating these molecular changes in future research may offer deeper insights into the mechanisms by which endometriosis contributes to ovarian cancer progression and prognosis.

This study investigated the prognosis of women with a history of surgical intervention for endometriotic ovarian cysts among those diagnosed with OCCC, a topic rarely addressed in the literature. Since this study does not compare the prognosis of surgically treated versus non-treated groups among patients with endometriomas, it is difficult to conclude that operated cases have a poorer survival rate compared to non-operated cases. Instead, considering the results of this study, which indicate a poor prognosis in individuals with a history of endometriotic ovarian cyst surgery, and the pathophysiological nature of endometriosis, which has the potential to transform into OCCC, physicians should closely monitor patients with endometriosis, particularly those with a surgical history of endometriosis. Given the poor prognosis of women with a surgical history of endometriotic ovarian cysts, these findings could be used in the future to identify patients at a high risk for OCCC.

This study has several limitations. First, as a retrospective, single-institution study with a relatively small sample size, the generalizability of our findings is limited. Previous studies have indicated that the incidence of OCCC varies by ethnicity, with a higher prevalence in Asians (26.2%) than in Caucasians (8.7%) [15]. Since all participants were of Asian descent, additional research, including large-scale, multi-center, and population-based studies with diverse ethnic representation, is needed to validate these conclusions. Second, access to past medical records, including sonographic findings and surgical records from external institutions, was incomplete, which may have impacted the accuracy of clinical data. Third, potential confounding factors, such as the extent of optimal cytoreductive surgery and the administration of adjuvant chemotherapy, should be considered when interpreting the prognostic implications of OCCC. While these factors may influence outcomes, our study did not control for all possible confounders in the statistical analysis, highlighting the need for future studies to incorporate adjusted models. Additionally, the lack of long-term follow-up data restricts our ability to assess overall survival trends beyond the study period.

Despite these limitations, our study has notable strengths. Unlike previous research that primarily examined the diagnostic and prognostic differences between endometriosis-associated and non-endometriosis-associated OCCC, this study specifically investigates the prognosis of patients with endometriosis-related OCCC who also had a history of endometriosis surgery. This distinction may provide valuable clinical insights into the potential impact of prior surgical interventions on disease outcomes. Furthermore, our study contributes to the growing body of evidence examining the relationship between endometriosis and ovarian cancer, underscoring the need for individualized treatment strategies based on patient history.

## 5. Conclusions

Although endometriosis did not affect the prognosis of women with OCCC, those with endometriosis-related OCCC were diagnosed at an earlier stage and at a younger age. A history of endometriotic ovarian cyst surgery did not affect the detection of OCCC. However, women with a history of endometriotic ovarian cyst surgery have poor survival outcomes. With more detailed information on the types of surgery, the stage of endometriosis, and objective measurements of adhesion levels in OCCC surgery, further research is needed to establish a definitive correlation between surgical history and the future prognosis of OCCC. Potential hypotheses for this relationship include, e.g., the impact of surgical-induced adhesions on disease progression or the role of surgical interventions in altering the tumor microenvironment. Additionally, investigating the detrimental effects of specific surgical techniques on ovarian function and exploring the interactions between surgical history and inflammatory responses may provide deeper insights. Even though additional studies are required, this study is significant in highlighting the potential impact of endometriotic ovarian surgery on the future prognosis of OCCC.

## Figures and Tables

**Table 1:** Basic characteristics of women with non-endometriosis-associated OCCC and women with both OCCC and endometriosis.

	Non-Endometriosis-Associated OCCC (*N* = 141)	Endometriosis-Related OCCC (*N* = 118)	*p*-Value
Age (years)	53.95 ± 9.71 *	45.68 ± 7.98 *	**<0.001**
Stage			**0.046**
Early (Stage I/II)	86 (62.77%)	88 (75.21%)	
Late (Stage III/IV)	51 (37.23%)	29 (24.79%)	
Cancer Grade **			0.203
1	1 (0.71%)	0 (0.0%)	
2	29 (20.71%)	26 (22.03%)	
3	93 (66.43%)	85 (72.03%)	
4	17 (12.14%)	6 (5.08%)	
5	0 (0.0%)	1 (0.85%)	
Ovary mass location			0.606
Right	28 (19.86%)	24 (20.34%)	
Left	24 (17.02%)	15 (12.71%)	
Bilateral	88 (62.41%)	79 (66.95%)	
Not known	1 (0.71%)	0 (0.0%)	
Breast cancer history	4 (2.84%)	2 (1.69%)	0.543
Endometrial cancer history	3 (2.13%)	5 (4.24%)	0.412
Residual disease			0.441
None	97 (69.29%)	91 (77.12%)	
<0.5 cm	18 (12.86%)	13 (11.02%)	
<1 cm	9 (6.43%)	8 (6.78%)	
<2 cm	3 (2.14%)	0 (0%)	
≥2 cm	2 (1.43%)	1 (0.85%)	

OCCC, ovarian clear cell carcinoma. * Values expressed as mean ± standard deviation. ** Grade 1: nearly normal, Grade 2: some loosely packed abnormal cells, Grade 3: many abnormal cells, Grade 4: very few normal cells, and Grade 5: no normal cells. Bold data indicates statistically significant.

**Table 2:** Basic characteristics of endometriosis-related OCCC women with a history of surgical intervention for endometriotic ovarian cysts compared to those without surgical histories.

	Without Surgical History (*N* = 101)	With Surgical History (*N* = 17)	*p*-Value
Age (years)	45.84 ± 8.24 *	44.71 ± 6.35 *	0.59
Stage			0.339
Early (Stage I/II)	78 (77.23%)	10 (62.50%)	
Late (Stage III/IV)	23 (22.77%)	6 (37.50%)	
Cancer Grade **			0.974
2	22 (21.78%)	4 (23.53%)	
3	73 (72.28%)	12 (70.59%)	
4	5 (4.95%)	1 (5.88%)	
5	1 (0.99%)	0 (0.0%)	
Ovary mass location			0.249
Right	18 (17.82%)	6 (35.29%)	
Left	13 (12.87%)	2 (11.76%)	
Bilateral	70 (69.31%)	9 (52.94%)	
Breast cancer history	1 (0.99%)	1 (5.88%)	0.667
Endometrial cancer history	4 (3.96%)	1 (5.88%)	1.000
Residual disease			0.081
None	79 (78.22%)	12 (70.59%)	
<0.5 cm	10 (9.90%)	3 (17.65%)	
<1 cm	8 (7.92%)	0 (0%)	
<2 cm	0 (0%)	0 (0%)	
≥2 cm	0 (0%)	1 (5.88%)	

* Values expressed as means ± standard deviation. ** Grade 1: nearly normal, Grade 2: some loosely packed abnormal cells, Grade 3: many abnormal cells, Grade 4: very few normal cells, and Grade 5: no normal cells.

**Table 3:** Comparison of CA-125 levels at the time of diagnosis and prognosis between non-endometriosis-associated OCCC and endometriosis-related OCCC.

Variable	Non-Endometriosis-Associated OCCC	Endometriosis-Related OCCC	*p*-Value
	(*N* = 141)	(*N* = 118)	
CA-125	104.20 (29.90, 347.70) *	80.70 (32.40, 247.90) *	0.32
Prognosis (expire or hopeless discharge status)			0.226
Event N ^a^ (%)	34 (24.11%)	21 (17.80%)	
Event ^a^/1000 PYRs	5.31	4.06	
Unadjusted HR (95% CI)	Ref	0.71 (0.42–1.23)	

**^a^** Event: expire or hopeless discharge status. OCCC, ovarian clear cell carcinoma; PYRs, person years; HR, hazard ratio; CI, confidence interval. * Values expressed as the median (Q1,Q3).

**Table 4:** Comparison of CA-125 levels at the time of diagnosis and prognosis between women with endometriosis-related OCCC and a history of surgical intervention for endometriotic ovarian cysts and those without surgical histories.

Variable	Without Surgical History	With Surgical History	*p*-Value
	(*N* = 101)	(*N* = 17)	
CA-125	77.40 (32.10, 241.60) *	84.00 (40.80, 259.35) *	0.629
Prognosis (expire or hopeless discharge status)			**0.008**
Event N ^a^ (%)	15 (14.85%)	6 (35.29%)	
Event ^a^/1000 PYRs	3.17	13.36	
Unadjusted HR (95% CI)	Ref	3.48 (1.39–8.69)	

^a^ Event: expire or hopeless discharge status. HR, hazard ratio; CI, confidence interval; PYRs, person years. * Values expressed as the median (Q1, Q3). Bold data indicates statistically significant.

## Data Availability

The raw data supporting the conclusions of this article will be made available by the authors without undue reservation.
